# How Do Blended Biochemistry Classes Influence Students’ Academic Performance and Perceptions of Self-Cognition?

**DOI:** 10.3389/fpsyg.2022.843392

**Published:** 2022-02-24

**Authors:** Guijie Ren, Peiyue Zhuang, Xianren Guan, Keli Tian, Jiping Zeng

**Affiliations:** ^1^Department of Biochemistry and Molecular Biology, School of Basic Medicine, Shandong University, Jinan, China; ^2^College of Liberal Arts, University of Minnesota Twin Cities, Minneapolis, MN, United States; ^3^Institute of Agricultural Remote Sensing and Information, Heilongjiang Academy of Agricultural Sciences, Harbin, China

**Keywords:** blended classroom, biochemistry, academic performance, self-cognition, semi-flipped

## Abstract

The flipped classroom is becoming a popular new instructional model in higher education capable of increasing student performance in higher-order learning outcomes. However, the success of a flipped classroom model depends on various supporting elements, and it may not be appropriate for all students and courses. In this study, a new blended Biochemistry classroom model based on Massive Open Online Courses (MOOC) and a “semi-flipped” environment was applied to Biochemistry instruction of Nursing and Clinical Medicine majors. The students’ academic performance and perceptions of self-cognition were used to assess the blended Biochemistry classroom. Students who participated in the blended classroom model achieved higher academic performance (*p* < 0.01) and reported a significant improvement in their perceptions of self-cognition (*p* < 0.05) compared to the control group. Moreover, the effectiveness of the blended Biochemistry classroom on the small size class (Nursing major) was stronger than on the large size class (Clinical Medicine major).

## Introduction

With the development of computers and internet, advances in information technology have enabled innovations in teaching and learning with new pedagogical methods and modes including flipped classrooms, Massive Open Online Courses (MOOC), and blended classes (Crouch and Mazur, 2001; Kuehl et al., 2019). Traditional instruction involving face-to-face lectures no longer meets the demands of students.

### The Flipped Classroom and Its Advantages

The earliest flipped classroom was originated from Mazur’s creation of peer instruction in 1990s (Crouch and Mazur, 2001). Flipped classroom, also known as inverted classroom, is becoming an increasingly popular pedagogical method in higher education. A scholar defined the complete FLIPPED model, consisting of Flexible Environment, Learning Culture, Intentional Content and Professional Educator, Progressive Networking Activity, Engaging and Effective Learning Experiences and Diversified and Seamless Platforms (Chen et al., 2014). Flipped classroom involves students undertaking activities that include watching teaching videos, self-study lectures, reading textbooks and other materials, and completing online assignments and quizzes prior to face-to-face classes. Face-to-face class then involves students discussing topics, solving problems, broadening knowledge applications, and generally undertaking activities designed to promote higher order thinking, all under the guidance from a teacher (Davies et al., 2013; Kuehl et al., 2019). As such, events that have traditionally taken place inside the classroom now take place outside the classroom and vice versa, hence the name ‘flipped’ (Burke and Fedorek, 2017).

According to the revised Bloom’s taxonomy of educational objectives (Anderson et al., 2000), from bottom to top, a pyramid representation indicating the cognitive process dimension covers lower-order thinking skills “Remember,” “Understand,” and higher-order thinking skills “Apply,” “Analyze,” “Evaluate,” and “Create.” The lower-order thinking skills are easier to acquire than the higher-order thinking skills. All active learning pedagogy should facilitate higher learning outcomes according to the revised Bloom’s taxonomy.

The flipped classrooms are based on the principle of active learning whereby students are actively or experientially involved in the learning process (Burke and Fedorek, 2017). It is generally thought that active learning is associated with higher student motivation, confidence, and critical thinking skills (Chiu and Cheng, 2017). Many teachers believe that students learn best when they are engaged actively in the learning process (Tse et al., 2019). Compared to traditional classes, flipped classrooms have been shown to increase students’ performance in higher-order learning outcomes such as the ability to apply, analyze, evaluate and create (Gaughan, 2014). To sum up, the “flipped classroom” is regarded as a potential and extraordinary learning method that engages students in applying their leaning knowledge and conducting higher order thinking, rather than receiving direct teaching instruction (Davies et al., 2013; Flumerfelt and Green, 2013). Through the flipped classroom, teachers can develop meaningful activities to stimulate the students to engage in higher order thinking (Kim et al., 2014).

Flipped classrooms also provide flexibility, customization, and accessibility for students learning (Waha and Davis, 2014). In this regard, self-paced learning has been the most acceptable characteristic and extensively studied (Chen et al., 2019).

### The Challenge of Flipped Classroom

Despite the many advantages of flipped classrooms described above, their success relies on the extent to which students have prepared before engaging with the in-class activities. One of the participating instructor observed that about 25% of the students had not watched online lectures in his previous experiment (Kim et al., 2014). Obviously, without preparation meant low participation in a group work. Previous study also revealed that some of the students still had difficulty adopting the flipped classroom approach because of their residual passive learning habits from the traditional classroom, where learning required less proactive effort (Chen et al., 2014). For example, in the out-of-class learning activities, students may fail to schedule their time to watch the videos and comprehend the learning content owing to their lack of self-regulation. In this circumstance, they are likely to fail to effectively learn in the following in-class activities (Mason et al., 2013). In other words, some students lack self-discipline and self-management and did not like the flipped learning model (Awidi and Paynter, 2019). To meet this challenge, some previous researchers proposed a self-regulated flipped classroom approach to help students schedule their out-of-class time to effectively read and comprehend the learning content before class, such that they are capable of interacting with their peers and teacher in class for in-depth discussion (Lai and Hwang, 2016).

### The Emerge of Blended Classroom

The self-regulated flipped classroom approach resolved the problem that some students lack self-discipline and self-management to some extent. However, Biochemistry is complicated and it is difficult for students to self-study prior to face-to-face class. Given the complexity of Biochemistry, the passive learning habits of students from the traditional classroom, and probable students anxiety regarding a totally new pedagogy, a blended “semi-flipped” classroom of Biochemistry was proposed based on the integration of traditional and flipped classroom models with MOOC.

MOOC, another pedagogical method that has emerged in recent years, has been described as a disruptive force in education because it challenges the traditional lecture-based class and decentralizes the education experience in a learner centered way (Robinson, 2016). In addition to the traditional study materials such as teaching videos, digital textbooks, problem sets and syllabus, MOOC provides interactive user forums that help build an online community for students and teachers. Due to its open online contents and massive participants, the MOOC mode is rapidly increasing, and many characteristic MOOC projects have appeared all over the world. For example, Chinese University MOOC on iCourse was jointly launched by iCourse and Netease Cloud in 2011 (Li and Zhang, 2014), immediately attracting the interest of not only academics and students within higher education, but also that of secondary students and teachers (Brahimi and Sarirete, 2015). With the continuing success of MOOC, it becomes a platform on which study materials are delivered for implementing flipped classrooms.

Semi-flipped classroom contains both the traditional teaching section (helping students to understand the difficult knowledge and master the key points) and the flipped classroom section (facilitating higher learning outcomes and developing the higher-order skills of students). The semi-flipped model contained advantages of both traditional and flipped learning. In our study, this new blended classroom model based on MOOC and a semi-flipped environment was implemented in Biochemistry teaching of Nursing and Clinical Medicine majors of higher education. To the best of our knowledge, it is the first reported application of the blend semi-flipped model for a Biochemistry course in medical higher education.

Therefore, in this study, a blended classroom model based on MOOC and a semi-flipped environment was proposed for remedy the main obstacles presented by the implementation of the flipped classroom. Moreover, several research questions were investigated to evaluate the effectiveness of the proposed model:

1. Can the blended classroom model improve the students’ academic performance in comparison with the traditional classroom?
2. Can the blended classroom model improve the students’ perceptions of self-cognition in comparison with the traditional classroom?

## Methodology

### Biochemistry Course Information and Ethics Approval

Biochemistry is a sophomore-level course at Cheeloo College, Shandong University. The content of Biochemistry mainly includes the metabolism and regulation of the carbohydrate, fat and amino acids, and the transformation of some other non-nutrients of human body. Also, it introduces the pathogenesis of some diseases which are resulted from abnormal material metabolism. It is important and difficult for the sophomore students because this is the first time for them to contact the clinical cases. Biochemistry is a compulsory course for undergraduate students of all five majors offered. Of these possible majors, two were involved in our study: Nursing and Clinical Medicine. Ethics approval was granted by the Ethics Committee of Shandong University prior to commencement of the study.

### Participants

Clinical Medicine is the largest major containing 245 students who are divided into 2 classes: Clinical Medicine-1, and Clinical Medicine-2, while the Nursing major is the smallest one containing 31 students. All 276 students enrolled across the two majors in Autumn 2020 were informed about the study and signed the consent forms. The 247 students enrolled in the same two majors in Autumn 2019 and who received traditional instruction served as the control groups. All students of these two grades were accommodated in the same campus with a similar learning environment, including the availability of internet and computer, the same content of learning, the same lecturers and professors, etc., which means that except for different periods of time and teaching model, other conditions in grade 2019 are consistent with those in 2020. Participant demographics are shown in Table 1.

**Table 1:** Participant demographics.

	Nursing		CM-1		CM-2	
	Traditional	Blended	Traditional	Blended	Traditional	Blended
Male	2	1	49	52	49	50
Female	28	30	60	71	59	72
Total	30	31	109	123	108	122

*Traditional, the class instructed by traditional teaching model in 2019 as control group; Blended, the class instructed by blended teaching model in 2020 as experimental group. CM-1, Clinical Medicine-1; CM-2, Clinical Medicine-2*.

### Course Design Toward a Blended Classroom Based on MOOC and a “Semi-Flipped” Environment

Half of the usual 4 classes weekly allotted to Biochemistry according to the University timetable were allocated to online self-study. On the Chinese University MOOC platform, selected study materials were made available 1 week before the face-to-face class. These materials included videos (which could be watched repeatedly) made by the teachers themselves and designed to teach students the key knowledge points, PowerPoint lectures, and syllabus that could be downloaded for further study. Students were required to discuss the topics delivered for that week in an online forum that counted for their credits. For each online unit quiz, it could be done before or after face-to-face class, and students can make 3 attempts to earn a high score.

The face-to-face component was designed as a “semi-flipped” class. The first half of the class (50-min duration) was used for enhancing students’ retention of the knowledge and testing their understanding. This was achieved with 10 choice questions using Rain Class APP (a cellphone software). This part of the class was still predominantly led by the teacher in a manner similar to traditional Biochemistry classes. After a 10-min interval, another 50-min class was a student-centered design which contained various of higher-order activities, including problem analysis and solving, clinical case discussions, and/or other applications of the knowledge, chosen based on the specific knowledge being examined. To complete these activities, students were divided into groups of 5–6 participants. 10 min. before the end of the class, one student from 3 to 4 groups was randomly chosen by the teacher to present the discussion results of their groups to the whole class.

### Instruments

MOOC platform was used to upload the study materials which were requisite for student self-study prior to the face-to-face class. The platform system log was used to evaluate students’ study process and performance, including video watching time, discussion forum participation, and the score of online unit quizzes, etc. The teacher can check the students’ learning logs and performance and then conduct some discussion based on any misunderstanding or high-error-rate questions in the online forum. Students’ learning logs and performance would be counted for the course credit to encourage students overcoming idleness, promoting proactive effort, improving self-discipline and self-management, and developing active learning habit.

Students’ academic performance was assessed using the total (final) score of the Biochemistry course, consisting of 4 sections: 30 points for online self-study, comprising 10 points for participation in the online discussion forum and 20 points for the average of all online unit quizzes; 10 points for the average of test results conducted with Rain Class APP in face-to-face classes; 10 points for reflecting the behavior in the class activities; and 50 points for the final exam. Each section’s points were added to obtain the total score. This assessment method reflected process evaluation, encouraging students worked hard at ordinary time.

Perceptions of self-cognition of students were also used to assess the instructional effectiveness of blended Biochemistry classes compared to traditional classes. We defined the self-cognition by student’s self judgement on the level of their own quality or ability to perform certain tasks. A survey of self-cognition consisting of 6 statements on a five-item Likert scale was sent out twice to all participants, before and after the study. The 6 statements were shown as below,

S1: I have the ability to learn proactively.

S2: I am a team player.

S3: I have question-based learning skills.

S4: I have the ability to speak publicly.

S5: I have the ability to learn cooperatively.

S6: I have the ability of time management.

In addition, 2 close-ended questions were involved in the second survey as follows: (a) How many hours did you use for Biochemistry study a week? (b) Do you prefer the blended classroom or the traditional classroom in future?

As an incentive for student participation, extra credit of an additional 2 points were given on the final exam. In total, 276 usable completed responses were collected. The response rate for the student survey reached 100%.

### Data Analysis

To evaluate students’ academic performance, independent *t*-test and One-way ANOVA were conducted to show differences in the final scores achieved using traditional and blended classes within each major, and across the two majors, respectively. To quantitatively analyze students’ perceptions, a five-item Likert scale was used, ranging from 5 to 1 with “strongly agree,” “agree,” “neutral,” “disagree,” and “strongly disagree,” respectively. Data are presented as mean ± SD, and One-way ANOVA and dependent *t*-test were conducted appropriately. All statistical analyses were conducted using IBM SPSS statistical software, version 22.0; *p* < 0.05 and *p* < 0.01 were considered as significant difference and extremely significant difference, respectively.

## Results

### Academic Performance

**Figure 1:**
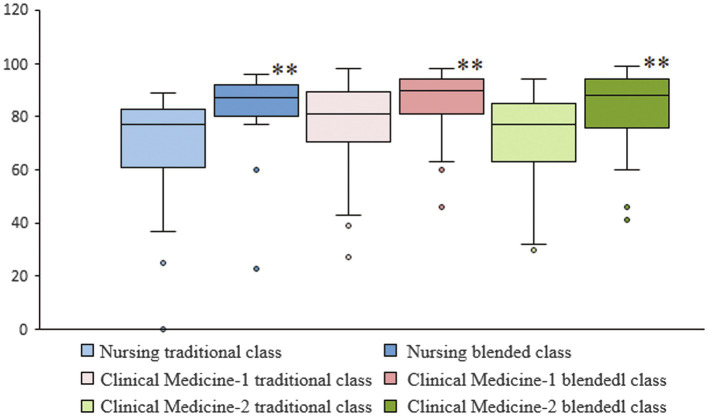
Box plots showing differences in total scores between traditional and blended classes within each major. Extremely significant difference (*p* < 0.01) within any of the three classes are designated by “^**^”.

To assess academic performance, students’ total scores were analyzed. The number of participants, mean scores, and standard deviations (SD) in the two majors are shown in Table 2. Box plots of independent *t*-test results between traditional and blended classes within each major are shown in Figure 1. Extremely significant differences (*p* < 0.01) were found between the traditional and blended classes for each of the two majors.

**Table 2:** Means and standard deviations (SD) of total score in different majors and groups.

	Nursing		CM-1		CM-2	
	Traditional	Blended	Traditional	Blended	Traditional	Blended
Number	30	31	109	123	108	122
Mean	73.53	83.04	77.63	86.23	73.37	84.66
SD	15.15	14.71	14.52	10.74	14.63	11.83

*Traditional, the class instructed by traditional teaching model in 2019 as control group; Blended, the class instructed by blended teaching model in 2020 as experimental group. CM-1, Clinical Medicine-1; CM-2, Clinical Medicine-2*.

One-way ANOVA was applied to analyze differences in academic performance of traditional or blended classes across the two majors. The ANOVA summary is shown in Table 3. For traditional classes, there was no statistically significant difference in total score means across the two majors [*F* (2, 244) = 2.538, *p* = 0.08]. Similarly, no statistically significant difference was observed in blended classes [*F* (2, 273) = 1.038, *p* = 0.356].

**Table 3:** One-way ANOVA summary of academic performance results across the two majors for traditional and blended classes.

	Source	SS	df	MS	*F*	*P*
Traditional class	Between groups	1088.85	2	544.43	2.538	0.08
	Within groups	52343.97	244	214.52		
	Total	53432.82	246			
Blended class	Between groups	281.41	2	140.71	1.038	0.356
	Within groups	36205.81	273	135.60		
	Total	36487.22	275			

*Traditional class, the class instructed by traditional teaching model in 2019 as control group; Blended class, the class instructed by blended teaching model in 2020 as experimental group*.

### Students’ Perceptions Before the Beginning of the Study

Results of the quantitative and one-way ANOVA analyses of students’ perceptions to six statements using a five-item Likert scale prior to the beginning of the study are shown in Tables 4, 5, respectively. The scores of students’ perceptions range from 3.01 to 3.61. The ANOVA results showed no statistically significant differences across the three classes for each of 6 statements [*F* (2, 273) = 0.552, *p* = 0.577], [*F* (2, 273) = 0.041, *p* = 0.960], [*F* (2, 273) = 0.195, *p* = 0.823], [*F* (2, 273) = 0.461, *p* = 0.631], [*F* (2, 273) = 1.245, *p* = 0.289], and [*F* (2, 273) = 0.236, *p* = 0.790], respectively.

**Table 4:** Means (±SD) of calculation results for the responses to 6 statements in the three classes before the beginning of the study.

	S1	S2	S3	S4	S5	S6
	Mean (SD)	Mean (SD)	Mean (SD)	Mean (SD)	Mean (SD)	Mean (SD)
Nursing	3.61	3.23	3.32	3.19	3.58	3.35
(*n* = 31)	(±0.919)	(±0.921)	(±0.909)	(±1.046)	(±1.177)	(±0.915)
CM-1	3.48	3.27	3.22	3.04	3.31	3.48
(*n* = 123)	(±0.765)	(±0.884)	(±0.869)	(±0.924)	(±0.820)	(±0.827)
CM-2	3.45	3.24	3.21	3.01	3.32	3.45
(*n* = 122)	(±0.757)	(±0.906)	(±0.869)	(±0.937)	(±0.816)	(±0.871)

*S1: I have the ability to learn proactively. S2: I am a team player. S3: I have question-based learning skills. S4: I have the ability to speak publicly. S5: I have the ability to learn cooperatively. S6: I have the ability of time management. CM-1, Clinical Medicine-1; CM-2, Clinical Medicine-2*.

**Table 5:** One-way ANOVA summary of students’ perceptions across the three classes before the beginning of the study.

	Source	SS	df	MS	*F*	*P*
S1	Between groups	0.67	2	0.337	0.552	0.577
	Within groups	152.23	273	0.611		
	Total	152.90	275			
S2	Between groups	0.07	2	0.033	0.041	0.960
	Within groups	193.62	273	0.807		
	Total	193.69	275			
S3	Between groups	0.30	2	0.149	0.195	0.823
	Within groups	182.74	273	0.765		
	Total	183.04	275			
S4	Between groups	0.82	2	0.412	0.461	0.631
	Within groups	213.68	273	0.894		
	Total	214.50	275			
S5	Between groups	1.89	2	0.947	1.245	0.289
	Within groups	178.04	273	0.761		
	Total	179.93	275			
S6	Between groups	0.35	2	0.174	0.236	0.790
	Within groups	175.25	273	0.736		
	Total	175.60	275			

*S1: I have the ability to learn proactively. S2: I am a team player. S3: I have question-based learning skills. S4: I have the ability to speak publicly. S5: I have the ability to learn cooperatively. S6: I have the ability of time management*.

### Students’ Perceptions After the Completion of the Blended Biochemistry Course

After completion of the blended Biochemistry course, a second survey containing the same 6 statements as the pre-course survey was conducted to evaluate the effectiveness of the blended classroom model on students’ self-cognition. Results of the quantitative and one-way ANOVA analyses of students’ perceptions are shown in Tables 6, 7, respectively. The scores of students’ perceptions range from 3.53 to 4.19. Interestingly, the score of perceptions (most of them >4) is significant higher in Nursing class than that in Clinical Medicine classes after completion of Biochemistry teaching. The one-way ANVOA analysis showed that there was no statistically significant difference in S1, S3, and S6 of students’ perceptions across the three classes [*F* (2, 273) = 1.048, *p* = 0.352], [*F* (2, 273) = 0.129, *p* = 0.879], [*F* (2, 273) = 0.448, *p* = 0.639], respectively. However, for S2, S4, and S5, there were significantly differences across the three classes [*F* (2, 273) = 3.804, *p* = 0.024], [*F* (2, 273) = 3.444, *p* = 0.034], and [*F* (2, 273) = 4.181, *p* = 0.016; Table 7], respectively.

**Table 6:** Means (±SD) of calculation results for the responses to 6 statements in the three classes after completion of the blended Biochemistry course.

	S1	S2	S3	S4	S5	S6
	Mean (SD)	Mean (SD)	Mean (SD)	Mean (SD)	Mean (SD)	Mean (SD)
Nursing	4.06	4.00	3.61	4.03	4.19	3.84
(*N* = 31)	(±0.892)	(±0.950)	(±1.022)	(±0.983)	(±0.792)	(±0.934)
CM-1	3.83	3.54	3.53	3.60	3.71	3.68
(*N* = 123)	(±0.568)	(±0.872)	(±0.782)	(±0.853)	(±0.852)	(±0.839)
CM-2	3.89	3.55	3.53	3.57	3.72	3.68
(*N* = 122)	(±0.788)	(±0.835)	(±0.824)	(±0.900)	(±0.875)	(±0.862)

*S1: I have the ability to learn proactively. S2: I am a team player. S3: I have question-based learning skills. S4: I have the ability to speak publicly. S5: I have the ability to learn cooperatively. S6: I have the ability of time management. CM-1, Clinical Medicine-1; CM-2, Clinical Medicine-2*.

**Table 7:** One-way ANOVA summary of students’ perception across the three classes after the completion of a blended Biochemistry course.

	Source	SS	df	MS	*F*	*P*
S1	Between groups	1.30	2	0.651	1.048	0.352
	Within groups	144.61	273	0.621		
	Total	145.91	275			
S2	Between groups	5.72	2	2.858	3.804	0.024^*^
	Within groups	177.31	273	0.751		
	Total	183.03	275			
S3	Between groups	0.18	2	0.089	0.129	0.879
	Within groups	162.40	273	0.697		
	Total	162.58	275			
S4	Between groups	5.48	2	2.739	3.444	0.034^*^
	Within groups	184.50	273	0.795		
	Total	189.98	275			
S5	Between groups	6.12	2	3.059	4.181	0.016^*^
	Within groups	170.43	273	0.731		
	Total	176.55	275			
S6	Between groups	0.67	2	0.333	0.448	0.639
	Within groups	172.48	273	0.744		
	Total	173.15	275			

S1: I have the ability to learn proactively. S2: I am a team player. S3: I have question-based learning skills. S4: I have the ability to speak publicly. S5: I have the ability to learn cooperatively. S6: I have the ability of time management. “^*^”, significant difference.

To determine which two classes present the significant difference, Tukey’s HSD post-hoc test was utilized to further analyze differences in perceptions across the three classes. The test indicated significant differences in perceptions between Nursing and CM-1 class, or between Nursing and CM-2 class for S2 (*p* = 0.006 and 0.005, respectively), S4 (*p* = 0.009 and 0.007, respectively), and S5 (*p* = 0.003 and 0.004, respectively).

**Figure 2:**
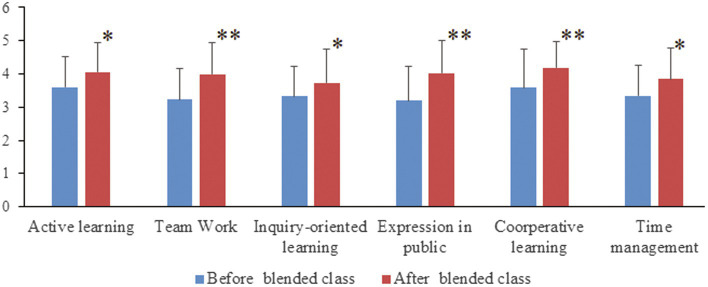
Comparisons of Nursing students’ perceptions for each statement before and after completing the Biochemistry blended course. Following completion of the blended Biochemistry course, there were significant improvements in students’ perceptions for all six statements compared to pre-course perceptions. “^*^”, significant difference; “^**^”, extremely significant difference.

**Figure 3:**
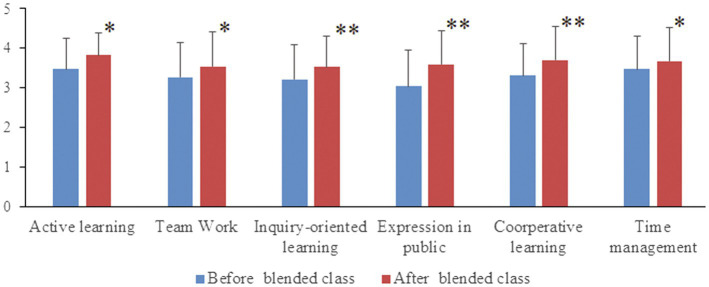
Comparisons of Clinical Medicine (CM-1 class) students’ perceptions for each statement before and after completing the Biochemistry blended course. Following completion of the blended Biochemistry course, there were significant improvements in students’ perceptions for all six statements compared to pre-course perceptions. “^*^”, significant difference; “^**^”, extremely significant difference.

**Figure 4:**
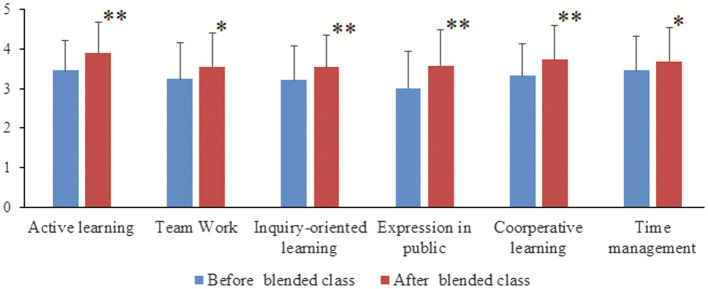
Comparisons of Clinical Medicine (CM-2 class) students’ perceptions for each statement before and after completing the Biochemistry blended course. Following completion of the blended Biochemistry course, there were significant improvements in students’ perceptions for all six statements compared to pre-course perceptions. “^*^”, significant difference; “^**^”, extremely significant difference.

Further analysis of any differences in student perceptions for each major before and after blended classes was undertaken using a dependent *t*-test. Significant or extremely significant differences for all 6 statements were found across each of the 3 classes (Figures 2–4).

## Discussion

### Blended Biochemistry Classroom and MOOC

MOOC platform is easier for students to attain learning resources and apply their abilities to practical application (Shu, 2018). Implementation of flipped classroom model is believed to enable teachers to better engage students and facilitate them in active learning, resulting in better learning outcomes (Freeman et al., 2014). Thus the MOOC platform can help teachers to implement the flipped or semi-flipped classroom. In this study, the new blended classroom based on MOOC and a “semi-flipped” environment was first applied to Biochemistry instruction for students majoring in Nursing and Clinical Medicine, which has not been reported in medical teaching to the best of our knowledge. The performance of students’ self-study on the MOOC was recorded in the database of the platform system log. It was used for the students’ process evaluation which was counted for their credits. This approach was benefit to instill in the students a habit of active learning, which ultimately improved their academic performance and perceptions.

### Academic Performance

The findings of this study showed that students who participated in the blended classroom model displayed a higher academic performance as measured by total score for the course compared to those receiving the traditional classroom instruction. This was consistent with previous study that most students showed positive attitude to flipped learning with better academic achievement and higher course satisfaction compared to the traditional class (Yu and Wang, 2016).

In the face-to-face classes, the teacher initially reinforced and summarized the key knowledge points contained in the self-study activities, followed by a quiz to test students’ understanding. Then students participated in higher-order study activities including analyzing phenomena, solving problems and discussing clinical cases. These activities were expected to invoke active learning among the students (Biswadeep and Chayna, 2015; Das and Sarkar, 2015). Teacher’s reinforcement and summary of knowledge, as well as higher-order study activities were the possible reasons for students to acquire higher academic performance.

Some researchers also found that use of the flipped classroom format resulted in STEM students spending more time engaging with the course content, leading to higher exam grades (Talley and Scherer, 2013; Sezer, 2017). In our study, participants’ responses to the second survey showed that the average time of studying Biochemistry was 10 h weekly, greater than the scheduled 6 h a week.

In addition to improvements in academic performance with the blended classroom format, there were no statistically significant differences across the two majors with traditional instruction (*p* = 0.08), or with the blended classroom (*p* = 0.356; Table 3). A possible explanation for this is that the blended classroom provides more study materials, equally available to all students, albeit the students were instructed by different teachers in the two majors.

### Students’ Perceptions

In this study, perceptions of self-cognition were used to reflect students’ higher-order thinking skills. And all students’ self-cognitions were improved in this study. It was consistent with previous study (Hui, 2018). The participants agreed that “this model is conducive to cultivate their abilities of active and self-learning.” Additionally, this model fostered students’ collaboration and communication skills, improving their problem-solving abilities (Karjanto and Simon, 2019).

With traditional classroom instruction, easier processes are generally performed in the classroom while more difficult processes are usually delegated to the students *via* assignments at home. Consequently, when students need the assistance of teachers the most, hands-on assistance and guidance are lacking. In contrast, the flipped or semi-flipped classroom model reverses the time and place of these learning activities, with easier learning objectives and acquisition of the lower-order thinking skills delegated to self-learning prior to face-to-face classes, and learning activities aiding in acquiring the higher-order thinking skills performed in the classroom. Thus, the lower-order skills are acquired before classes, while higher-order skills are developed in class where assistance is provided through the teacher’s guidance and peer collaborative learning activities (Fischer and Haenze, 2019; Lo and Hew, 2019). This may explain the significant improvement in the students’ perceptions of self-cognition reflecting the higher-order thinking skills after completion of the blended Biochemistry course compared to the perceptions before the blended Biochemistry course began.

In addition, there were statistically significant differences in S2, S4 and S5 of students’ perceptions between Nursing and Clinical Medicine classes after the completion of blended course (Table 7). A possible explanation for this is that Nursing class was a small size class, containing 31 students, whereas CM-1 and CM-2 are large size classes with both more than 100 students. In the small size class, it’s more convenient for students to acquire assistance from the teacher.

Moreover, in the second survey of this study, when students were asked whether they preferred the blended classroom or traditional classroom in future, 89% of students in Nursing major and 62% of student in Clinical Medicine major preferred the blended classroom, respectively. These results indicated that the students in small size classes were more satisfied with the blended classroom than those of large size classes.

## Conclusion

The blended Biochemistry classroom based on MOOC and a semi-flipped environment was effective on the improvements of both students’ academic performance and outcomes of self-cognition. Although there was no significant academic performance difference between two majors, from the improvement of self-cognition, the results showed that the effectiveness on small size class was stronger than that on the large size class. And the blended Biochemistry classroom was better accepted by students of small size class.

The limitations of this study have to be noted. As this study was administered by the course instructor, there was a certain element of coercion and undue influence on student participation despite the compensation being 2 extra credits. This choice of experiment format was to learn from previous studies’ low response rate of 25% (Kim et al., 2014). And luckily, the added concern for unfair grading due to the choice of participation was eliminated by the fact that 100% of students took part and earned the 2 extra points.

The result of this study will provide some insight for the instruction of medical higher education. It may be implicated in other courses of the university. In the future, similar studies could be done on students of different majors and different courses, or have the experiment run for an extended period of time.

## Data Availability Statement

The original contributions presented in the study are included in the article/supplementary material, further inquiries can be directed to the corresponding author.

## Ethics Statement

The studies involving human participants were reviewed and approved by The Ethics Committee of Shandong Universtiy. The patients/participants provided their written informed consent to participate in this study.

## Author Contributions

All the authors contributed to the study design and approved the final version of the manuscript for submission. XG collected and analyzed the data. GR and KT analyzed the data and drafted the manuscript. PZ revised the manuscript, defined the important factors, and modified the language of revised manuscript. JZ designed the questionnaires, and proof read the manuscript.

## Funding

The authors acknowledge the financial support from Undergraduate Education Reform Project of Shandong province (No. M2018B351), the Undergraduate Teaching Reform Project of Cheeloo Medical college, Shandong University (No. qlyxjy201907), and Course Ideology and Politics Special Project (No. 2020JF49).

## Conflict of Interest

The authors declare that the research was conducted in the absence of any commercial or financial relationships that could be construed as a potential conflict of interest.

## Publisher’s Note

All claims expressed in this article are solely those of the authors and do not necessarily represent those of their affiliated organizations, or those of the publisher, the editors and the reviewers. Any product that may be evaluated in this article, or claim that may be made by its manufacturer, is not guaranteed or endorsed by the publisher.
